# PolyGR‐containing aggregates link with AD pathological hallmarks and autophagy pathway

**DOI:** 10.1002/alz70855_101865

**Published:** 2025-12-23

**Authors:** Huong T. Phuong, Rodrigo Francisco Tomas, Cemal Akamese, Ana Mijares, Shu Guo, Logan R. Bell, Svitlana Yegorova, Stefanie K. Ng, Jennifer L. Phillips, Alexandra N. Melloni, Olga Pletnikova, H. Brent Clark, Juan C. Troncoso, Bradley T. Hyman, Laura P. W. Ranum, Stefan Prokop, Lien Nguyen

**Affiliations:** ^1^ University of Florida, Gainesville, FL, USA; ^2^ Massachusetts General Hospital, Boston, MA, USA; ^3^ Johns Hopkins University School of Medicine, Baltimore, MD, USA; ^4^ University of Minnesota, Minneapolis, MN, USA; ^5^ MassGeneral Institute for Neurodegenerative Disease, Massachusetts General Hospital, Harvard Medical School, Charlestown, MA, USA

## Abstract

**Background:**

Alzheimer's disease (AD) is prevalent in people ≥ 65‐year‐olds, which counts up to ∼75% dementia cases worldwide. AD patient is characterized by progressive cognitive impairment, the accumulation of beta‐amyloid (Aβ) plaques and neurofibrillary hyperphosphorylated tau (pTau) tangles. Molecular mechanisms of most AD cases are not fully understood. We recently identified poly‐Glycine‐Arginine containing (polyGR+) aggregates that frequently accumulate in AD autopsy brains. We also identified interrupted *GGGAGA* repeat expansions (exp) in *CASP8* that produces polyGR+ protein accumulated in brain tissue from expansion AD carriers. Specific *CASP8*‐*GGGAGA^exp^* variants are associated with increased AD risk. Previous studies suggested that impairment of autophagy‐lysosomal pathway, which maintains cellular protein homeostasis, play important role in AD pathogenesis. In this study, we examine if polyGR+ aggregates are associated with known AD pathological hallmarks (Aβ, pTau). We also study if polyGR+ aggregates are linked with disruption of autophagy‐lysosomal pathway.

**Method:**

We performed immunohistochemical staining for polyGR, Aβ and pTau using the hippocampal sections from 133 AD cases, 30 controls, and 15 primary age‐related tauopathy (PART) cases. We studied the levels of polyGR+ aggregate staining and Aβ and pTau deposition levels in the hippocampal regions. Double immunofluorescence (IF) staining of polyGR and autophagy‐lysosomal markers (p62 and LC3B) was performed to study if polyGR co‐aggregates with p62 and LC3B in AD autopsy brains.

**Result:**

Consistent to our previous study, frequent a‐polyGR positive aggregates are found in ∼50% AD autopsy brains but not in controls and PART cases (*p* <0.0001). PolyGR+ signal accumulates as cytoplasmic or nuclear puncta in CA regions, subiculum and presubiculum regions in the hippocampus of AD autopsy brains. We detect positive correlations of polyGR+ staining with Aβ (R^2^=0.2544, *p* = 0.0007) and pTau (R^2^ =0.2822, *p* = 0.0003), which is stronger than the correlation of Aβ and pTau in the hippocampus (R^2^=0.1047, *p* = 0.0366). PolyGR+ aggregates co‐localize with p62 and LC3B staining in polyGR+ AD brains.

**Conclusion:**

Our data demonstrates that polyGR+ aggregates are an important proteinopathological feature in AD autopsy brains. Additionally, our data suggests that co‐localization of autophagy and proteasome markers p62 and LC3B with polyGR+ aggregates could lead to dysregulation of autophagy‐lysosomal pathway, which contributes to disease pathogenesis.

